# Effects of garlic supplementation on components of metabolic syndrome: a systematic review, meta-analysis, and meta-regression of randomized controlled trials

**DOI:** 10.1186/s12906-023-04038-0

**Published:** 2023-07-22

**Authors:** Zhenyue Fu, Jiayu Lv, Xiya Gao, Haoran Zheng, Shuqing Shi, Xia Xu, Bingxuan Zhang, Huaqin Wu, Qingqiao Song

**Affiliations:** 1 Department of General Internal Medicine, Guang’anmen Hospital, China Academy of Chinese Medical Sciences, Beijing, China; 2 Beijing University of Chinese Medicine, Beijing, China; 3 Department of Cardiology, Guang’anmen Hospital, China Academy of Chinese Medical Sciences, Beijing, China

**Keywords:** Garlic, Metabolic syndrome, Systematic review, AMETA-analysis, Randomized controlled trials

## Abstract

**Background:**

Garlic (Allium sativum), the underground bulb of the *Allium genus*, has been consumed on Earth for thousands of years. Many clinical trials of garlic supplementation on components of metabolic syndrome (MetS) have emerged in recent years, but there is no consensus on the effect. This meta-analysis aimed at systematically evaluating the effect of garlic supplementation on components of MetS.

**Methods:**

In this meta-analysis, we searched Pubmed, Embase, Cochrane, Medline, Web of Science databases, and clinical trials online sites from inception to November 1, 2022, with language restrictions to English. We engaged participants > 18 years and eligible for the clinical diagnosis of MetS or those with metabolic disorders and garlic was the only intervention. Outcomes included waist circumference, and body mass index, triglycerides, total cholesterol, low-density lipoprotein cholesterol, high-density lipoprotein cholesterol, blood pressure, and fasting blood glucose. Meta-regression and subgroup analyses were conducted based on six covariates (total sample size, the mean age, the mean dose, the duration of intervention, the oral form of garlic, and the dietary intervention).

**Results:**

Results from 19 RCTs were included engaging 999 participants. Compared to placebo, garlic significantly reduced TG [SMD (95%CI) = -0.66 (-1.23, -0.09)], TC [SMD (95%CI) = -0.43 (-0.86, -0.01)], LDL [SMD (95%CI) = -0.44(-0.88, -0.01)], DBP [SMD (95%CI) = -1.33 (-2.14, -0.53)], BMI [SMD (95%CI) = -1.10(-1.90, -0.20)], and WC [SMD (95%CI) = -0.78(-1.09, -0.47)]. Meta-regression showed age and sample size are potential effect modifiers.

**Conclusion:**

According to the results of meta-analysis, the modulatory effect of garlic on some MetS components is evident. More high-quality, large-scale RCTs are needed to confirm iat based on the high heterogeneity and potential publication bias of the current data.

**Trial registration:**

https://www.crd.york.ac.uk/prospero/display_record.php?RecordID=373228, ID: CRD42022373228.

**Supplementary Information:**

The online version contains supplementary material available at 10.1186/s12906-023-04038-0.

## Introduction

Metabolic syndrome(MetS) is a condition of metabolic disorder in which central obesity, dyslipidemia, insulin resistance, and hypertension exist as a cluster [1]. As a breeding ground for various serious diseases (cardiovascular disease, type 2 diabetes, polycystic ovary syndrome) [2–4], the four major components of the MetS were previously named by scientists as the "deadly quartet" [5]. Pathophysiological mechanisms such as inflammation, endoplasmic reticulum stress, activation of the renin angiotensin aldosterone system, and dysbiosis of the intestinal flora are intertwined in the formation of MetS [6–9]. Regions around the world are witnessing the epidemic of MetS, with an adult prevalence of approximately 35% in the United States and 14% in China [10], and the number continues to grow at an alarming rate. Currently, no comprehensive treatment and management plan is available for MetS, and modification of dietary habits is a simple, feasible, and efficient intervention.

Garlic (Allium sativum), the underground bulb of the *Allium genus*, originated in Central Asia and the Mediterranean region thousands of years ago and is nowadays loved by people worldwide [11]. Many researchers hypothesize and verify that the high concentration of sulfur-containing bioactive compounds(alliin, allicin, S-allyl cysteine, and diallyl trisulfide) in its bulb can alleviate oxidative stress, apoptosis, inflammation, and vascular remodeling to ameliorate MetS [11, 12]. And the growing clinical evidence also suggests that the effect of garlic and its extracts on MetS [13]. A. A. Sangouni, et al. conducted an RCT finding that garlic lowered metabolic components, insulin resistance, fatty liver index and appetite in patients with MetS [14]. Meanwhile, a systematic review negated the lipid-lowering effect of garlic [15]. Therefore, no consistent conclusion has been reached and it is necessary to employ thorough meta-analysis on relevant RCTs to quantitatively evaluate the effect of garlic on MetS components.

## Materials and methods

This meta-analysis and systematic evaluation were performed in strict compliance with the Cochrane Handbook [16] and registered in PROSPERO (ID: CRD42022373228). The results were presented according to the 27 entries listed in the PRISMA checklist. (Supplementary Table S1) Literature retrieval, inclusion, data extraction, and quality evaluation were performed by two researchers simultaneously and independently. Any discrepancy was resolved through discussion or consulting a third researcher to reach a consensus.

### Information sources and retrieval strategy

Two members independently searched Pubmed, Embase, COCHRANE, Medline, and Web of science, with the time frame limited to the date of establishment to November 1, 2022, and the publication language limited to English. The search strategy used a combination of subject words plus free words, including garlic, allicin, metabolic syndrome, hypertension, hyperlipidemia, insulin resistance, waist circumference, and body mass index. (Supplementary Data 1) Additional searches were conducted at the National Institutes of Health (http://clinicaltrials.gov/) and the WHO International Clinical Trials Registry Platform (www.who.int/clinical-trials-registry-platform) to find ongoing clinical studies. References in relevant published systematic reviews or reviews were also scanned to reduce omissions.

### Eligibility criteria

The inclusion criteria for this study were strictly based on the PICOS principles and contained the following entries. 1) Participants: participants > 18 years and eligible for the clinical diagnosis of MetS or those with a state of disordered glucose, lipid, and blood pressure, the diagnostic criteria for MetS can be derived from any of the international authoritative organizations; 2) Intervention: garlic and its derivatives or extracts (row garlic, allicin, produced garlic powder, aged garlic) as the only intervention; 3) Control: placebo with no evidence to affect outcomes; 4) Outcomes: include one or more of the following indices: waist circumference (WC), and body mass index (BMI), triglycerides (TG), total cholesterol (TC), low-density lipoprotein cholesterol (LDL-c), high-density lipoprotein cholesterol(HDL-c), blood pressure (SBP, DBP), and fasting blood glucose (FBG); 5) Study: parallel or crossover RCTs.

Exclusion criteria were: 1) The control group received non-pharmacological therapies such as exercise; 2) Participants with heart disease, chronic kidney disease, gastrointestinal disorders, participants who are pregnant or breastfeeding, or participants who smoke or abuse alcohol; 3) Taking other medications that impact weight, blood pressure, lipids, and blood glucose; 4) Outcomes are not available; 5) Reviews, commentaries, conference abstracts, and case reports.

### Data extraction and quality evaluation

We entered the following information in the standardized data extraction form: 1) Basic information: first author's name, nationality, institution, and year of publication; 2) Baseline information: sample size, male/female ratio, mean age, health status, and baseline disease; 3) Trial information: the oral form of garlic, placebo composition, dose, duration of intervention and dietary intervention; 4) Outcomes: WC, BMI, TG, TC, LDL-c, HDL-c, FBG, SBP, DBP. 5) Trial process: randomization method, implementation of allocation concealment, blinded format.

Included studies were independently evaluated according to the criteria of the Cochrane Handbook (version 5.1.0). The evaluation entries included: the generation of random sequences, allocation concealment, blinding, selection bias, incomplete outcome information, selective reporting of outcomes, and other sources of bias. The risk of bias for each entry was evaluated as "low risk," "high risk," and "unclear risk".

### Data process and analysis

Data analysis was conducted according to the statistical guidelines referenced in the current version of the Cochrane Handbook. To avoid errors caused by unit inconsistency, we used Cohen's standardized mean difference (SMD) to evaluate the effect value [16]. The cut-off values of 0.2, 0.5, and 0.8 were used to divide SMD, corresponding to low, medium, and high effects. For data measured multiple times, the most recent data after the trial completion was selected. For studies with multiple intervention dose groups, the highest-dose group was selected. For presenting data results as means and standard deviations, they were transformed into means and standard deviations of pre- and post-intervention differences. For quartile data, they were transformed into mean and standard deviation format using the method developed by Hozo SP [17]. For crossover RCTs, the first-stage data were extracted to avoid the cumulative effects on the later results.

Data analysis was performed by Review Manager 5.4, Stata17 (StataCorp LP, College Station, US), and R 4.2.1. Data in this study were all continuous variables and effect sizes were presented as SMD and 95% CI. Low, medium, and high levels of heterogeneity were decided by the I^2^ statistic of 25%, 50%, and 75%. If I^2^ > 50%, significant heterogeneity was indicated, and the effect sizes were combined using a random-effects model. Subgroups were divided based on total sample size, the mean age of the intervention group, the mean daily dose of garlic, the duration of intervention, and the oral form of garlic. Meta-regression and subgroup analysis were performed to detect and elucidate the sources of high heterogeneity. Sensitivity analysis was performed to screen the RCT’s impact on the robustness of the results. Contour-enhanced funnel plot and Egger’s test were used to detect publication bias. In the Contour-enhanced funnel plot, the dark to light gray represents 90%, 95%, and 99% CIs, and the white area in the middle is the invalid interval. If most of the scatter falls in the white interval, we consider that no publication bias of negative results exists [18].

### Quality of evidence and GRADE approach

The evidence for each index of the meta-analysis was assessed based on the five entries (risk of bias, inconsistency, indirectness, imprecision, publication bias) listed in the GRADE (Grading of Recommendations Assessment, Development and Evaluation) handbook (https://gdt.gradepro.org/app/handbook/handbook.html) and classified into four levels: high quality, moderate quality, low quality, and very low quality [19]. This process was performed on the GRADEpro GDT online website (https://gdt.gradepro.org).

## Results

### Research screening and description

**Fig. 1:**
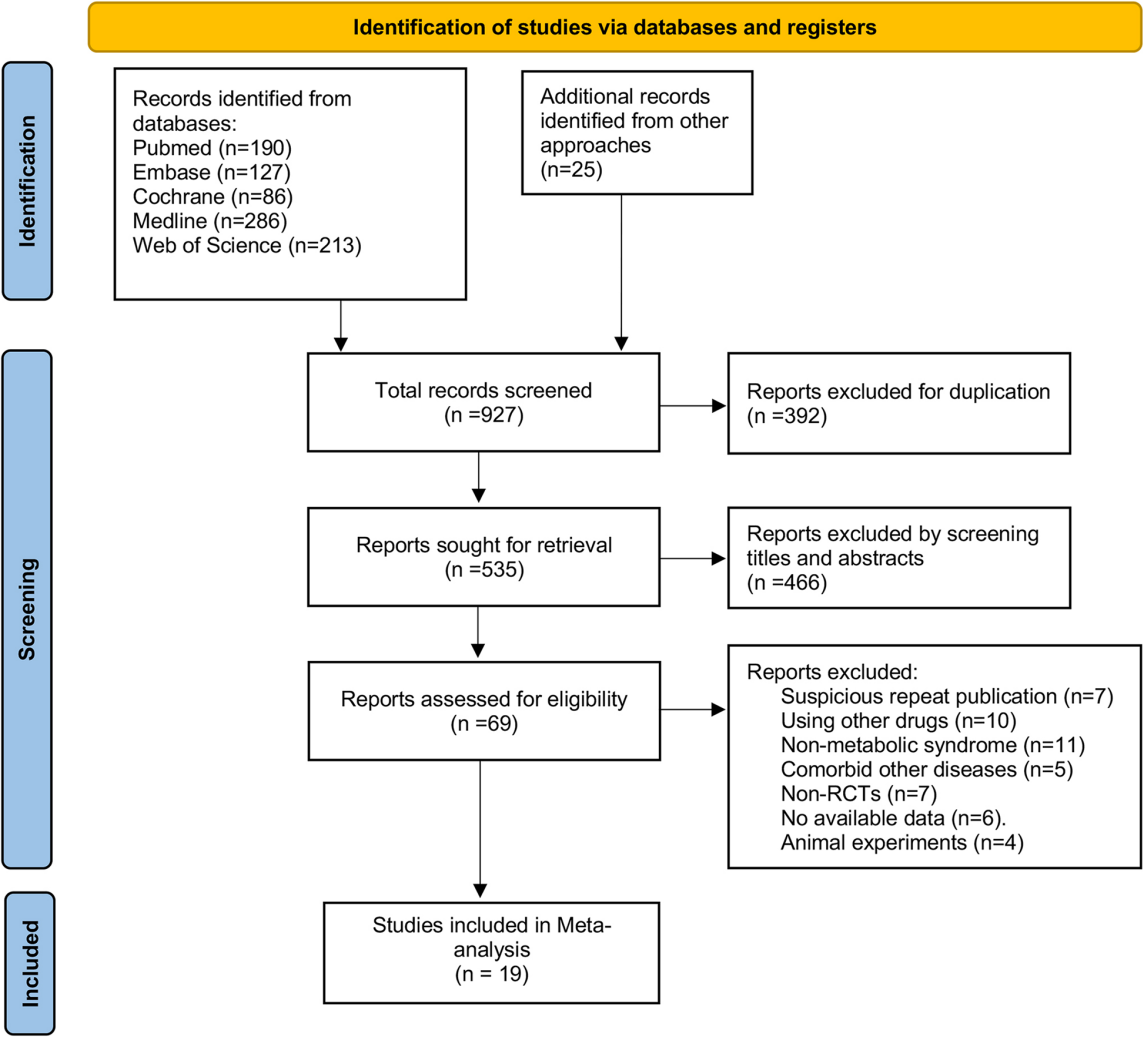
Flow chart of literature screening

A total of 927 papers were retrieved, and 392 duplications were removed. 466 irrelevant papers were excluded after reading the titles and abstracts. We read the full text of the remaining 69 articles, and 50 papers were excluded for various reasons. The remaining 19 papers were included in the meta-analysis (Fig. 1).

### Basic characterization and quality evaluation

**Fig. 2:**
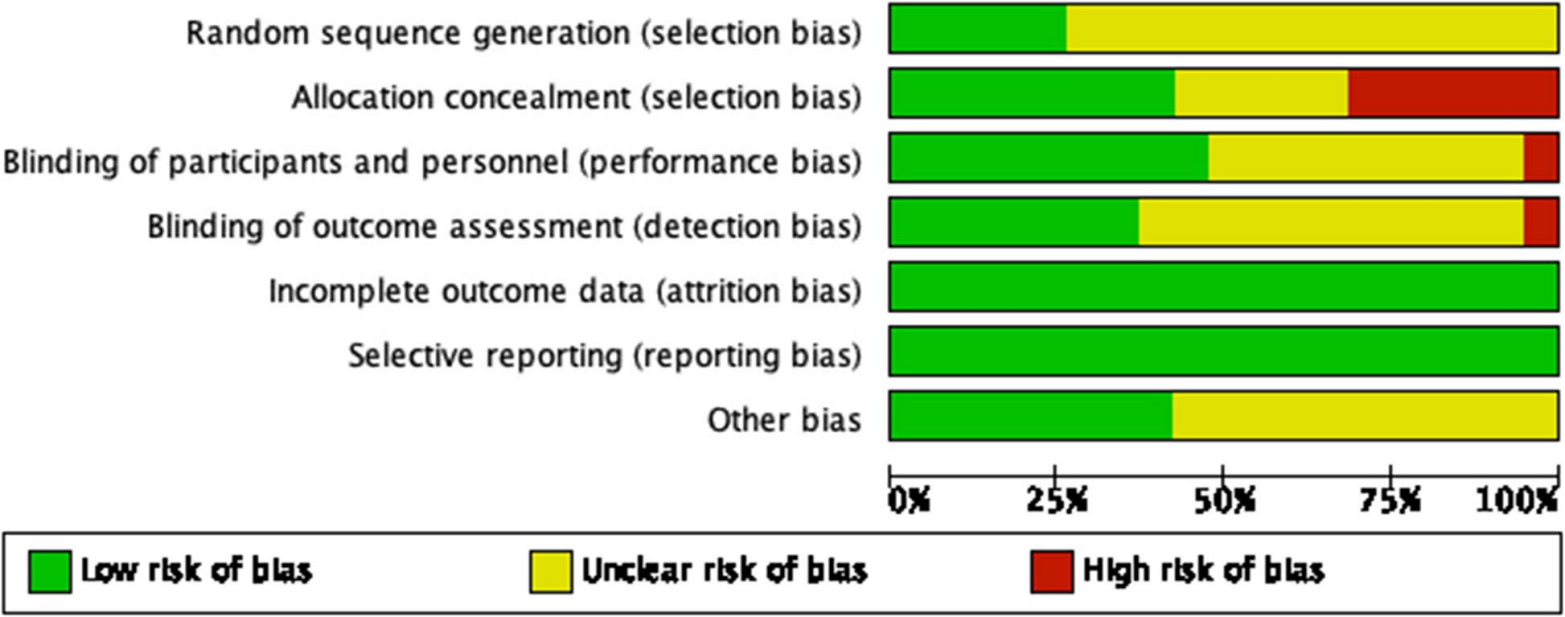
Risk of bias of assessment

A total of 19 RCTs were included [14, 20–37], including 17 in a parallel design [14, 20–34, 36] and 2 in a crossover design [35, 37]. The total number of participants was 999, including 497 in the treatment group versus 502 in the control group. The mean age fluctuated from 39 to 63. Eight studies were conducted in Asia [14, 21, 25, 28, 30, 31, 33, 34], five in North America [20, 24, 26, 27, 36], and three each in Oceania [29, 32, 35] and Europe [22, 23, 37]. Baseline diseases included hyperlipidemia, hypertension, MetS, and nonalcoholic fatty liver disease. One treatment group used raw garlic orally [21], three used aged garlic extract [28, 32, 37], one used processed garlic [25], and the remainder used garlic powder tablets. The intervention doses of garlic powder ranged from 188 to 2400 mg. The duration of each group ranged from 6 to 24 weeks (Table 1). The results of the quality evaluation of each RCT are shown below (Fig. 2).

**Table 1:** Basic information of the included randomized controlled trials

Author year	Country	Trial design	Sample		Mean age		Baseline disease	Intervention		Dosage	Duration	Outcomes
			T	C	T	C		T	C	mg/d	/w	
Adler, A. J.,1997 [20]	Canada	parallel	11	11	45.9	45.4	hyperlipidemia	GT	placebo	900	12	TG,TC,LDL,HDL
Aslani, N.,2016 [21]	Iran	parallel	27	28	45.3	39.3	hyperlipidemia	RG	none		8	BMI,TG,TC,LDL,HDL
Auer 1990 [22]	Germany	parallel	24	23	NR	NR	hypertension	GT	placebo	600	12	SBP,DBP
Byrne, DJ.,1999 [23]	UK	parallel	20	11	NR	NR	hyperlipidemia	GT	placebo	900	24	TG,TC,LDL,HDL
Gardner, CD.,2001 [24]	USA	parallel	16	18	50.2	51.6	hyperlipidemia	GT	placebo	999	12	TG,TC,LDL,HDL
Higashikawa, F., 2012 [25]	Japan	parallel	28	26	52	51.4	hyperlipidemia	PG	placebo	900	12	BMI,TG,TC,LDL,HDL,FBG
Isaacsohn, JL.,1998 [26]	USA	parallel	24	18	58	57.4	hyperlipidemia	GT	placebo	900	12	BMI,TG,TC,LDL,HDL,SBP,DBP
Jain, AK.,1993 [27]	USA	parallel	20	22	48	55	hyperlipidemia	GT	placebo	900	12	TG,TC,LDL,HDL,FBG,SBP,DBP
Jung, ES.,2014 [28]	Korea	parallel	28	27	50.13	50.83	hyperlipidemia	AGE	placebo		12	TG,TC,LDL,HDL
Kannar, D.,2001 [29]	Australia	parallel	19	22	52.6	57.4	hyperlipidemia	GT	placebo	800	12	TG,TC,LDL,HDL
Nakasone,Yasushi, 2013 [30]	Japan	parallel	19	21	58	59	hypertension	GT	placebo	188	12	SBP,DBP
Peleg, A.,2003 [31]	Israel	parallel	13	20	52.4	54.7	hyperlipidemia	GT	placebo		16	TG,TC,LDL,HDL
Riad, Karin,2018 [32]	Australia	parallel	23	26	62.8	61.9	hypertension	AGE	placebo	2400	12	WC,BMI,TG,LDL,HDL,FBG,SBP,DBP
Sangouni, Abbas A,2020 [33]	Iran	parallel	45	43	45.2	44.2	NAFLD	GT	placebo	1600	12	WC,BMI,TG,TC,LDL,HDL
Sangouni, Abbas A,2021 [14]	Iran	parallel	42	42	46.9	44.6	MetS	GT	placebo	1600	12	TG,TC,LDL,HDL
Sharifi, F.,2010 [34]	Iran	parallel	20	20	47.9	50.5	MetS	GT	placebo	1800	6	WC,BMI,TG,TC,HDL,FBG,SBP,DBP
Simons, LA.,1995 [35]	Australia	crossover	12	17	53.6	53.6	hyperlipidemia	GT	placebo	900	12	TG,TC,LDL,HDL,SBP,DBP
Superko, HR. 2000 [36]	USA	parallel	25	25	53	53	hyperlipidemia	GT	placebo	900	12	SBP,DBP
Valls, RM.,2022 [37]	Spain	crossover	32	34	53.7	52.7	hyperlipidemia	AGE	placebo	250	6	SBP,DBP

*T* Treatment group, *C* Control group, *NR*: not reported, *NAFLD* Non-alcoholic fatty liver disease, *MetS* Metabolic syndrome, *GT* Garlic tablet, *RG* Row garlic, *PG* Processed garlic, *AGE* Aged garlic extract, *WC* Waist circumference, *BMI* Body mass index, *TG* Triglycerides, *TC* Total cholesterol, *LDL-c* Low-density lipoprotein cholesterol, *HDL-c* High-density lipoprotein cholesterol, *FBG* Fasting blood glucose, *SBP* Systolic blood pressure, *DBP* Diastolic blood pressure

### Effect of garlic on the components of MetS

#### Effect of garlic on anthropometric indices

The results showed that BMI [SMD (95%CI) = -1.10(-1.90, -0.20), *p* < 0.05, I^2^ = 92.1%, 6 trials, 324 participants], and WC [SMD (95%CI) = -0.78(-1.09, -0.47), *p* < 0.05, I^2^ = 0.00, 3trials, 173 participants] were significantly lower in the treatment group (Table 2 and 3).

**Table 2:** Meta-analysis of the effect of garlic on WC

Study	SMD (95% CI)	Weight %
Riad, Karin., 2018 [32]	-0.78 (-1.22, -0.33)	48.82
Sangouni, Abbas Ali., 2021 [14]	-0.77 (-1.21, -0.34)	51.18
Sharifi, F., 2010 [34]	(excluded)	0
Overall (I-squared = 0.00, p=0.985)	-0,78 (-1.09, -0.47)	100

**Table 3:** Meta-analysis of the effect of garlic on BMI

Study	SMD (95% CI)	Weight %
Higashikawa, Fumiko., 2012 [25]	-0.35 (-0.89, 0.19)	20.96
Isaacsohn, JL., 1998 [26]	-0.08 (-0.69, 0.53)	20.5
Riad, Karin., 2018 [32]	-0.73 (-1.17, -0.29)	21.48
Sangouni, Abbas Ali., 2021 [14]	-0.35 (-0.78, 0.07)	21.58
Sharifi, F., 2010 [34]	-5.00 (-6.28, -3.72)	15.47
Aslani, N., 2016 [21]	(excluded)	0
Overall (I-squared = 92.1%, p=0.000)	1.10 (-1.99, -0.20)	100

#### Effect of garlic on blood lipids

In random effects model, we found that garlic supplementation was associated with significant reductions in TG [SMD (95%CI) = -0.66 [-1.23, -0.09], *p* < 0.05,15trials, 701 participants], TC [SMD (95%CI) = -0.43 (-0.86, -0.01), *p* < 0.05,14trials, 617 participants], and LDL [SMD (95%CI) = -0.44(-0.88, -0.01), *p* < 0.05, 14trials, 577 participants]. We also observed a slight rise of HDL in the treatment group [SMD (95%CI) = 0.1(-0.28, 0.48), *p* = 0.59, 15 trials, 701 participants] with no significance (Tables 4, 5, 6 and 7).

**Table 4:** Meta-analysis of the effect of garlic on TG

Study	SMD (95% CI)	Weight %
Adler, A. J., 1997 [20]	-3.15 (-4.44, -1.87)	5.11
Aslani, N., 2016 [21]	-0.54 (-1.07, 0.00)	7.03
Byrne, DJ., 1999 [23]	-0.09 (-0.82, 0.65)	6.57
Gardner, CD., 2001 [24]	-0.13 (-0.70, 0.44)	6.96
Higashikawa, Fumiko., 2012 [25]	-0.33 (-0.87, 0.21)	7.03
Isaacsohn, JL., 1998 [26]	-0.30 (-0.91, 0.32)	6.86
Jain, AK., 1993 [27]	0.10 (-0.50, 0.71)	6.88
Jung, ES.,2014 [28]	-0.40 (-0.93, 0.14)	7.04
Kannar, D., 2001 [29]	0.54 (-0.09, 1.17)	6.84
Peleg, A., 2003 [31]	0.90 (-0.14, 1.94)	5.77
Riad, Karin., 2018 [32]	-0.93 (-1.38, -0.48)	7.2
Sangouni, Abbas Ali., 2021 [14]	-3.19 (-3.83, -2.56)	6.82
Sangouni, Abbas Ali., 2020 [33]	-0.34 (-0.90, 0.22)	6.98
Sharifi, F., 2010 [34]	-2.35 (-3.17, -1.54)	6.37
Simons, LA., 1995 [35]	0.25 (-0.99, 0.49)	6.56
Overall (I-squared = 89.5%, p=0.000)	-0.66 (-1.17, -0.16)	100

**Table 5:** Meta-analysis of the effect of garlic on TC

Study	SMD (95% CI)	Weight %
Adler, A. J., 1997 [20]	-20.48 (-26.89, -14.08)	0.42
Aslani, N., 2016 [21]	-1.09 (-1.66, -0.52)	8.61
Byrne, DJ., 1999 [23]	-0.48 (-1.23, 0.26)	7.75
Gardner, CD., 2001 [24]	-0.25 (-0.83, 0.32)	8.59
Higashikawa, Fumiko., 2012 [25]	-0.83 (-1.38, -0.27)	8.66
Isaacsohn, JL., 1998 [26]	0.19 (-0.43, 0.80)	8.4
Jain, AK., 1993 [27]	-0.37 (-0.99, 0.24)	8.41
Jung, ES., 2014 [28]	-0.23 (-0.76, 0.30)	8.78
Kannar, D., 2001 [29]	-0.66 (-1.29, -0.03)	8.31
Peleg, A., 2003 [31]	0.20 (-0.79, 1.19)	6.55
Sangouni, Abbas Ali., 2021 [14]	-0.93 (-1.37, -0.49)	9.17
Sangouni, Abbas Ali., 2020 [33]	-0.16 (-0.72, 0.39)	8.67
Simons, LA., 1995 [35]	0.71 (-0.05, 1.47)	7.67
Sharifi, F., 2010 [34]	(excluded)	0
Overall (I-squared = 81.9%, p=0.000)	-0.43 (-0.86, -0.01)	100

**Table 6:** Meta-analysis of the effect of garlic on LDL

Study	SMD (95% CI)	Weight %
Adler, A. J., 1997 [20]	-9.33 (-12.34, -6.32)	1.74
Aslani, N., 2016 [21]	-0.99 (-1.55, -0.3)	8.52
Byrne, DJ., 1999 [23]	-0.31 (-1.05, 0.43)	7.72
Gardner, CD., 2001 [24]	-0.18 (-0.76, 0.39)	8.7
Higashikawa, Fumiko., 2012 [25]	-1.11 (-11.68, -0.53)	8.46
Isaacsohn, JL., 1998 [26]	0.22 (-0.39, 0.84)	8.3
Jain, AK., 1993 [27]	-0.39 (-1.01, 0.22)	8.3
Jung, ES., 2014 [28]	-0.05 (-0.58, 0.48)	8.66
Kannar, D., 2001 [29]	-0.87 (-1.51, -0.23)	8.16
Peleg, A., 2003 [31]	0.17 (-0.82, 1.16)	6.57
Sangouni, Abbas Ali., 2021 [14]	-0.75 (-1.18, -0.32)	9.04
Sangouni, Abbas Ali., 2020 [33]	0.07 (-0.48, 0.63)	8.55
Simons, LA., 1995 [35]	0.99 (0.20, 1.77)	7.51
Overall (I-squared = 83.0%, p=0.000)	-0.44 (-0.88, -0.01)	100

**Table 7:** Meta-analysis of the effect of garlic on HDL

Study	SMD (95% CI)	Weight %
Adler, A. J., 1997 [20]	-1.90 (-2.92, -0.88)	5.18
Aslani, N., 2016 [21]	0.51 (-0.03, 1.05)	7.1
Byrne, DJ., 1999 [23]	-0.37 (-1.12, 0.37)	6.29
Gardner, CD., 2001 [24]	0.05 (-0.52, 0.62)	6.97
Higashikawa, Fumiko., 2012 [25]	0.23 (-0.31, 0.76)	7.11
Isaacsohn, JL., 1998 [26]	0.41 (-0.21, 1.03)	6.79
Jain, AK., 1993 [27]	-0.14 (-0.75, 0.47)	6.84
Jung, ES., 2014 [28]	0.41 (-0.12, 0.95)	7.11
Kannar, D., 2001 [29]	-0.88 (-1.53, -0.24)	6.69
Peleg, A., 2003 [31]	-0.06 (-1.04, 0.93)	5.31
Riad, Karin., 2018 [32]	1.34 (0.86, 1.81)	7.33
Sangouni, Abbas Ali., 2021 [14]	1.04 (0.60, 1.49)	7.43
Sangouni, Abbas Ali., 2020 [33]	-0.04 (-0.59, 0.52)	7.04
Sharifi, F., 2010 [34]	-0.74 (-1.38, -0.10)	6.7
Simons, LA., 1995 [35]	1.00 (0.21, 1.79)	6.12
Overall (I-squared = 82.8%, p=0.000)	0.10 (-0.28, 0.48)	100

#### Effect of garlic on blood pressure

In nine RCTs (423 participants), we found garlic supplementation was associated with a dramatic decline of DBP [SMD (95%CI) = -1.33 (-2.14, -0.53), *p* < 0.05, I^2^ = 92.1%], but with a slight decline of SBP [SMD (95%CI) = -0.56(-1.58, 0.47), *p* = 0.29, I^2^ = 95.1%] (Tables 8 and 9).

**Table 8:** Meta-analysis of the effect of garlic on SBP

Study	SMD (95% CI)	Weight %
Auer, W., 1990 [22]	-0.69 (-1.28, -0.10)	12.02
Isaacsohn, JL., 1998 [26]	0.22 (-0.39, 0.84)	11.99
Jain, AK., 1993 [27]	0.15 (-0.46, 0.76)	12
Nakasone, Yasushi, 2013 [30]	-0.69 (-1.41, 0.03)	11.8
Riad, Karin., 2018 [32]	-1.23 (-1.70, -0.77)	12.2
Sharifi, F., 2010 [34]	5.81 (4.37, 7.26)	10.04
Simons, LA., 1995 [35]	-0.55 (-1.31, 0.20)	11.73
Superko, HR., 2000 [36]	-0.10 (-0.58, 0.38)	12.18
Valls, RM., 2022 [37]	-13.11 (-16.12, -10.10)	6.04
Overall (I-squared = 95.1%, p=0.000)	-0.56 (-1.58, 0.47)	100

**Table 9:** Meta-analysis of the effect of garlic on DBP

Study	SMD (95% CI)	Weight %
Auer, W., 1990 [22]	-0.89 (-1.49, -0.29)	12.28
Isaacsohn, JL., 1998 [26]	-0.42 (-1.04, 0.20)	12.23
Jain, AK., 1993 [27]	0.00 (-0.61,0.61)	12.27
Nakasone, Yasushi, 2013 [30]	-0.96 (-1.70, -0.22)	11.87
Riad, Karin., 2018 [32]	-0.98 (-1.43, -0.52)	12.65
Sharifi, F., 2010 [34]	-2.77 (-3.65, -1.89)	11.39
Simons, LA., 1995 [35]	0.00 (-0.74, 0.74)	11.86
Superko, HR., 2000 [36]	-0.69 (-1.19, -0.19)	12.55
Valls, RM.,2022 [37]	-18.40 (-22.58, -14.22)	2.9
Overall (I-squared = 92.1%, p=0.000)	-1.33 (-2.14, -0.53)	100

#### Effect of garlic on FBG

The effect of garlic on FBG was reported in five studies involving 214 individuals. According to the forest plot, garlic supplementation reduces FBG mildly [SMD (95%CI) = -0.26(-0.95, 0.44), *P* = 0.469, I^2^ = 85.3%] with no statistical significance (Table 10).

**Table 10:** Meta-analysis of the effect of garlic on FBG

Study	SMD (95% CI)	Weight %
Higashikawa, Fumiko., 2012 [25]	0.00 (-0.53, 0.53)	20.9
Jain, AK., 1993 [27]	-0.10 (-0.71, 0.51)	20.18
Riad, Karin., 2018 [32]	-0.39 (-0.83, 0.04)	21.83
Sharifi, F., 2010 [34]	-1.76 (-2.49, -1.02)	18.82
Simons, LA., 1995 [35]	0.99 (0.20, 1.77)	18.28
Overall (I-squared = 85.3%, p=0.000)	-0.26 (-0.95, 0.44)	100

### Meta-regression and subgroup analysis

**Fig. 3:**
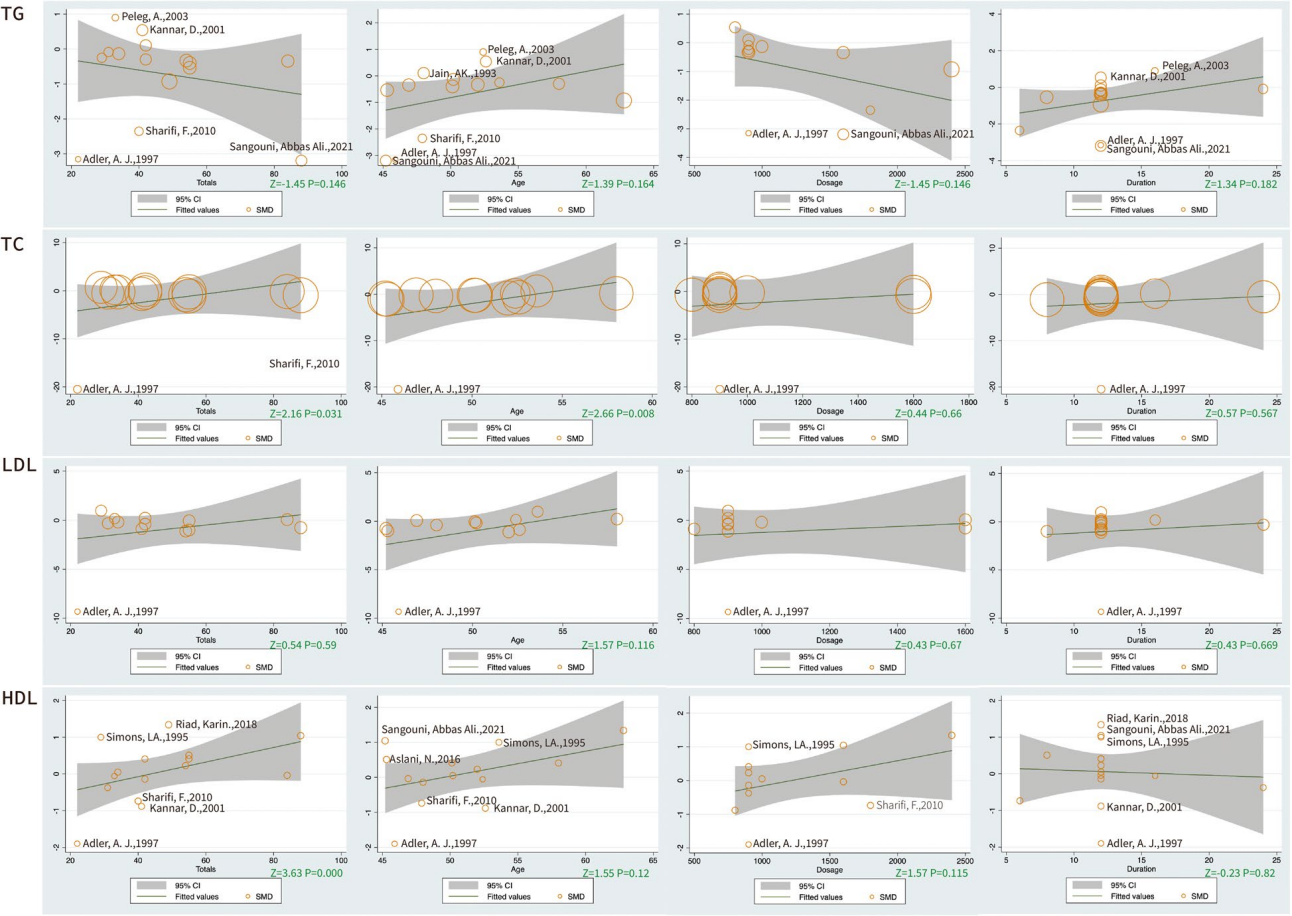
The results of meta-regression

Based on the high heterogeneity of the serological and anthropometric indices, we grouped covariates (potential effect modifiers) such as total sample size, the mean age, mean daily dose, duration of intervention, the oral form of garlic, and the dietary intervention that might affect the results. Indices that included more than 10 RCTs were analyzed by meta-regression based on these covariates. The results show that TC may be positively modulated by age (Z = 2.16, *P* < 0.05). Both TC (Z = 2.66, *P* < 0.05) and HDL (Z = 3.63, *P* < 0.05) showed an ascending trend as the sample size increased. However, there was no significant difference in the benefits of different garlic intervention doses for each index (Fig. 3).

To further explore the effect of potential covariates in participants with MetS, we did subgroup analysis for TG, TC, LDL, HDL, SBP, and DBP. Dividing the sample size by 50, we found thatin the larger sample trial (≥ 50), TG, TC, LDL, SBP, and DBP reduced significantly. In participants younger than 50 years old, we found TG, TC, LDL reduced significantly. To our surprise, during shorter interventions (< 10 weeks), TG, TC, LDL, SBP, and DBP showed significant decreases. Only the elevated HDL seemed associated with longer interventions. We found that row garlic and aged garlic may be more effective than produced garlic for their significant effect on the reduction of TC, LDL, SBP, DBP and the elevation of HDL. Strikingly, we found that under the advised diet, garlic played a weak role in regulating blood sugar and blood lipids (Table 11).

**Table 11:** Subgroup analysis of the included randomized controlled trials

	**TG**			**TC**			**LDL**		
	**Number**	**Combined Effect Value**	**Heterogeneity**	**Number**	**Combined Effect Value**	**Heterogeneity**	**Number**	**Combined Effect Value**	**Heterogeneity**
		SMD (95%CI) mmol/L	I^2^		SMD (95%CI) mmol/L	I^2^		SMD (95%CI) mmol/L	I^2^
**overall**	15	-0.66(-1.17, -0.16)*	89.50%	14	-0.43(-0.86, -0.01)*	81.90%	13	-0.44(-0.88, -0.01)*	83.00%
**Totals**									
≥ 50	5	-0.83(-1.08, -0.58)*	85.90%	5	-0.65(-1.02, -0.01)*	58.90%	5	-0.56(-1.02, -0.11)*	73.40%
< 50	10	-0.41(-0.62, -0.2)*	93.70%	9	-0.35(-1.07, 0.38)	85.70%	8	-0.46(-1.19, 0.27)	86.30%
**Age**									
≥ 50	8	-0.29(-0.5, -0.08)*	64.80%	7	-0.18(-0.55, 0.2)	58.70%	7	-0.15(-0.65, 0.35)	76.20%
< 50	7	-1.02(-1.28, -0.77)*	93.60%	7	-0.93(-1.76, -0.09)*	88.80%	6	-0.93(-1.71, -0.15)*	87.90%
**Dosage**									
≥ 1000	4	-1.41(-1.7,-1.13)*	94.40%	3	-0.53(-1.32, 0.19)	78.10%	2	-0.36(-1.16, 0.45)	80.90%
< 1000	8	-0.17(-0.4, 0.06)	74.10%	8	-0.47(-1.17, 0.22)	86.60%	8	-0.63(-1.36, 0.1)	88.00%
**Duration**									
≥ 10w	13	-0.51(-0.69, -0.34)*	89.50%	13	-0.37(-0.82, 0.07)	81.90%	12	-0.40(-0.86, 0.07)	
< 10w	2	-1.09(-1.54, -0.64)*	92.50%	2	-1.09(-1.66, -0.52)*		1	-0.99(-1.55, -0.43)*	83.30%
**Dietary intervention**									
none		-1.82(-3.66, 0.03)	91.90%	3	-10.22(-29.81, 9.37)	97.30%	2	-4.70(-13.54, 4.14)	96.90%
regular		-0.40(-0.70, -0.11)	44.30%	5	-0.55(-0.90, -0.21)	44.70%	5	-0.54(-0.97, -0.12)	64.40%
advised		-0.46(-1.61, 0.69)	94.30%	6	-0.16(-0.67, 0.35)	74.40%	6	-0.07(-0.62, 0.48)	77.70%
**Oral form**									
row	1	-0.54(-1.07, 0.00)		1	'-1.09(-1.66, -0.53)		1	'-0.99(-1.55, -0.43)	
aged	2	-0.68(-1.20, -0.17)	54.30%	1	'-0.23(-0.76, 0.30)		1	'-0.05(-0.58, 0.48)	
produced	12	-0.69(-1.36, -0.01)	91.60%	13	'-0.40(-0.90, 0.10)	83.40%	11	'-0.45(-0.97, 0.07)	84.60%
	**HDL**			**SBP**			**DBP**		
	**Number**	**Combined Effect Value**	**Heterogeneity**	**Number**	**Combined Effect Value**	**Heterogeneity**	**Number**	**Combined Effect Value**	**Heterogeneity**
		SMD (95%CI) mmol/L	I^2^		SMD (95%CI) mmol/L	I^2^		SMD (95%CI) mmol/L	I^2^
**overall**	15	0.1(-0.28, 0.48)	82.80%	9	-0.56(-1.58, 0.47)	95.1	9	-1.33(-2.14, -0.53)*	92.10%
**Totals**									
≥ 50	5	0.45(0.08, 0.82)*	61.50%	2	-6.52(-19.26, 6.23)	95.10%	2	-9.42(-26.77, 7.94)	98.50%
< 50	10	-0.09(-0.66, 0.47)	86.10%	7	0.27(-0.71, 1.25)	93.50%	7	-0.83(-1.39, -0.26)*	81.70%
**Age**									
≥ 50	8	0.33(-0.16, 0.81)	80.10%	6	-1.51(-2.65, -0.37)*	94.10%	6	-1.52(-2.62, -0.43)*	93.30%
< 50	7	-0.16(-0.79, 0.46)	86.00%	3	1.64(-0.92, 4.20)	97.00%	3	-1.19(-2.61, 0.23)	92.30%
**Dosage**									
≥ 1000	4	0.42(-0.47, 1.31)	91.40%	2	2.25(-4.65, 9.16)	98.80%	2	-1.23(-2.21, -0.26)*	92.30%
< 1000	8	-0.15(-0.64, 0.34)	76.60%	7	-1.08(-2.03, -0.12)*	92.30%	7	-1.83(-3.59, -0.07)*	92.10%
**Duration**									
≥ 10w	13	0.14(-0.28, 0.55)	83.10%	7	-0.42(-0.85, 0.01)	73.10%	7	-0.59(-0.90, -0.29)*	46.30%
< 10w	2	-0.10(-1.32, 1.12)	88.30%	2	-3.60(-22.14, 14.94)	99.20%	2	-10.45(-25.76,4.86)	98.10%
**Dietary intervention**									
none	3	-0.92(-1.69, -0.16)	65.00%	2	2.53(-3.85, 8.90)	98.50%		-1.80(-3.65, 0.04)	91.70%
regular	6	0.42(-0.02, 0.86)	74.60%	3	-3.82(-6.62, -1.03)	97.40%		-4.33(-7.18, -1.48)	97.30%
advised	6	0.26(-0.36, 0.88)	82.50%	4	-0.23(-0.62, 0.16)	34.90%		-0.54(-0.90, -0.19)	20.80%
**Oral form**									
row	1	0.51(-0.03, 1.05)	-	-	-	-	-	-	-
aged	2	0.88(-0.03, 1.79)	84.60%	2	-7.07(-18.71, 4.56)	98.30%	2	-9.56(-26.63, 7.52)	98.50%
produced	12	-0.07(-0.49, 0.35)	80.30%	7	0.41(-0.48, 1.29)	91.90%	7	'-0.78(-1.35, -0.22)	81%

^*^p < 0.05, statistically significant, *CI* Confidence interval, *TG* Triglycerides, *TC* Total cholesterol, *LDL-c* Low-density lipoprotein cholesterol, *HDL-c* High-density lipoprotein cholesterol, *SBP* Systolic blood pressure, *DBP* Diastolic blood pressure

### Sensitivity analysis and publication bias

To assess the robustness and reliability of the meta-analysis results, we performed a sensitivity analysis by removing the RCTs one by one. The results showed that removing any studies of TC, LDL, HDL, and DBP did not affect the results. While the robustness of TG may be affected by the data of Sangouni Abbas Ali and the robustness of SBP may be affected by the data of Riad Karin. While removing trials that may affect the results, we found the direction of combined statistics didn’t change (Supplementary Figure S1). In addition, we performed the trim and filling method for TG and SBP and found no censoring and addition.

**Fig. 4:**
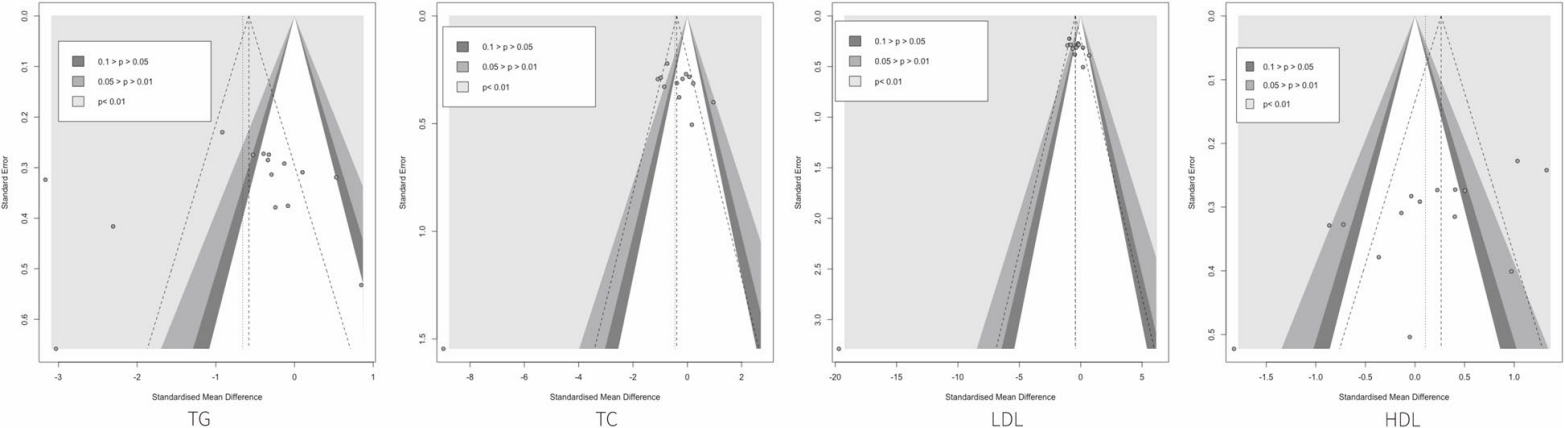
The results of publication bias

We used contour-enhanced funnel plots and Egger’s tests to detect the presence of publication bias. Most of the indices are symmetrically distributed, but we find asymmetry in the funnel plot of TG. However, most of its scatter fell in the right white invalid interval. We used Egger’s tests to avoid the possibility of a statistical Type I error with low test efficacy and find no publication bias of TG (*p* > 0.05) and potential publication bias of HDL(*p* < 0.05) (Fig. 4).

### Quality of evidence

Although the RCTs we included were all high-grade evidence, their quality declined with reporting bias and publication bias. The GRADE approach showed moderate-quality evidence for major indices, but low-quality evidence for the HDL due to publication bias (Table 12).

**Table 12:** Quality of evidence based on GRADE approach

Certainty assessment							№ of patients		Effect	Certainty	Importance
№ of	Study design	Risk of bias	Inconsistency	Indirectness	Imprecision	Other considerations	Treatment	Control	Absolute		
study									(95% CI)		
TG	Randomized trials	Serious	Not serious	Not serious	Not serious	None	362	339	SMD 0.66 SD lower	⨁⨁⨁◯	important
15									(1.17 lower to 0.16 lower)	Moderate	
TC	Randomized trials	Serious	Not serious	Not serious	Not serious	None	320	297	SMD 0.43 SD lower	⨁⨁⨁◯	important
14									(0.86 lower to 0.01 lower)	Moderate	
LDL	Randomized trials	Serious	Not serious	Not serious	Not serious	None	300	277	SMD 0.44 SD lower	⨁⨁⨁◯	important
13									(0.88 lower to 0.01 lower)	Moderate	
HDL	Randomized trials	Serious	Not serious	Not serious	Not serious	Publication bias strongly suspected	362	339	SMD 0.1 SD higher	⨁⨁◯◯	important
15									(0.28 lower to 0.48 higher)	Low	
SBP	Randomized trials	Serious	Not serious	Not serious	Not serious	None	206	217	SMD 0.56 SD lower	⨁⨁⨁◯	important
9									(1.58 lower to 0.47 higher)	Moderate	
DBP	Randomized trials	Serious	Not serious	Not serious	Not serious	None	206	217	SMD 1.33 SD lower	⨁⨁⨁◯	important
9									(2.14 lower to 0.53 lower)	Moderate	

*CI* Confidence interval, *SMD* Standard mean difference

## Disscusion

### Summary of evidence

MetS was first proposed in 1988, and its content has gradually been clarified over decades of development [5]. Currently, the WHO Diabetes Group, the US National Cholesterol Education Program: Adult Treatment Panel III, and the International Diabetes Federation Consensus Group formed a consensus that obesity and metabolic disorders of glucose, lipid, and blood pressure together contribute to MetS [38–40]. Nowadays, there is no comprehensive management for MetS, and improving lifestyle and dietary habits may be the best way to prevent and manage it [41, 42]. Lack of intervention will lay down the risk of CVD and diabetes [2]. In recent years, research has been emerging on food supplementation and plant-food-derived bioactive to intervene in MetS and its components [43]. Among them, garlic is a popular daily spicy condiment, and researchers are curious and enthusiastic about its potential to improve the human metabolic profile.

For an in-depth, extensive, and comprehensive review of the effect of garlic on the MetS components, we retrieved 19 RCTs (including 999 participants) from 10 countries by strictly limiting inclusion and exclusion criteria. We consumed garlic and its extract(row garlic, produced garlic powder, aged garlic) and placebo in the treatment and control groups and found garlic supplementation can modulate anthropometric indices (BMI, WC) and lipids (TG, TC, LDL), and lower DBP in MetS. Meta-regression showed that most indices wouldn’t be regulated by mean daily dose, duration of intervention, the oral form of garlic, and the dietary intervention. During subgroup analysis, we found lipid indices fall more in the older age group and prolonged intervention does not produce better modulatory effects of metabolic indices.

### Overview of underlying mechanisms

The pharmacological activity of garlic depends on the abundance of non-volatile sulfur-containing compounds such as alliin and S-allyl cysteine in the cytoplasm of its bulb, which accounts for about 3.5% of the weight of raw garlic and 80% of the total sulfur-containing compounds [44]. After the destruction process (squeezing and cutting), organosulfur compounds(allicin and S-allylcysteine) are catalyzed by alliinase to produce allicin and jointly play a role in regulating blood lipids, blood pressure, and blood sugar [45]. However, studies have shown that allicin is produced after 6 s of squeezing and cutting in raw garlic, and after digestion in the intestine with oral administration of processed garlic, and the process may be affected by the gastrointestinal environment (stomach acid, protease). Therefore, processed garlic may be less effective than raw garlic [46]. In the vitro model of metabolic syndrome, organosulfur compounds extracted from garlic can increase the abundance of acidophilus in the intestinal flora to improve insulin sensitivity and increase the production of taurine to upregulate PPARγ and CPT1A in the liver to promote β-oxidation of fatty acids [47]. Studies have also demonstrated that organosulfur compounds may synergistically exert various inhibitory effects in the process of cholesterol synthesis in the liver, such as inhibiting key enzymes in cholesterol synthesis (HMG-CoA reductase, cholesterol 7α-hydroxylase, fatty acid synthase) [15, 48, 49]. Meanwhile, allicin can also regulate lipid metabolic signals and modulate metabolic organs. In animal models with high-fat diet, allicin can up-regulate the expression of lipolytic genes (lipoprotein lipase and adipose triglyceride lipase), down-regulate the expression of fat degradation genes (fatty acid synthase, PPARγ), and promotes browning of white adipose tissue [50, 51]. For hypertension, garlic and its derivatives can alleviate oxidative stress by inhibiting the activity of ROS and NADPH oxidase [52]. Sulfur-containing compounds can produce H_2_S after erythrocyte action, and allicin can mediate the upregulation of angiotensin II receptors and eNOS, both of which have been shown to dilate blood vessels and lower blood pressure [53, 54]. Moreover, allicin can modulate insulin concentrations in STZ-induced diabetic rats with a dose-dependent response [55]. And garlic extract can bind to the druggable region of DPP-4 to inhibit its activity, exerting a hypoglycemic effect similar to that of DPP-4 inhibitors (selegiline) [56].

### Comparison with previous studies

There are no meta-analyses exploring the efficacy of garlic in treating MetS, but studies of garlic for a single MetS component (lipids, blood pressure, blood glucose) have been widely conducted. The benefits of garlic in lowering total cholesterol, blood pressure, and blood glucose are clear, but different findings existed for other lipid parameters [57–59]. The earliest meta-analysis showed that garlic reduced TC by 9% [60]. In subsequent large-scale meta-analyses, garlic showed significant reductions in TC and TG, but slight reductions in HDL and LDL [61, 62]. These inconsistent results may be related to insufficient participant samples and inconsistent baseline disease. In a recent meta-analysis investigating the association between garlic intake and cardiovascular disease, garlic could exert better lipid-regulating effects in CVD participants at low intervention times, which is consistent with the conclusions we obtained [63]. Nowadays, there is no comprehensive management for MetS, and improving lifestyle and dietary habits may be the best way to prevent and manage it. All evidence proves that daily garlic supplementation can be a potential modulator of metabolic disorders.

### Limitations

Undeniably, the present meta-analysis has potential limitations. The first is the high heterogeneity, we conducted subgroup analysis and meta-regression, but the source of heterogeneity still cannot be pinpointed completely. Based on our current evidence, sample size and age may be the potential components influencing heterogeneity. Secondly, the sensitivity analysis identified RCTs that could affect the robustness of the results, but the removal of the RCT did not change the direction of the effect and the trim and filling method suggested reliable results. Third, Egger’s tests showed the presence of publication bias for HDL. Fourth, according to the available information, we cannot conduct a further meta-analysis on the oral form of garlic. And a lack of consistency in garlic's origin and extraction process may also influence the results. Finally, based on the low to moderate level of evidence from the GRADE approach, we cannot draw a robust quantitative conclusion yet.

### Implications for future research

Based on the limitations encountered in this study, we make the following recommendations for subsequent research in this area. First, we suggest that RCTs should be reported strictly according to the standardization in the CONSORT statement [64] so that they can be evaluated and interpreted in subsequent studies. Second, more high-quality and large-scale meta-analyses are needed to improve the credibility of the outcomes. The possibility of risks in the Cochrane Handbook (version 5.1.0) should be reduced during the study design process. Studies focusing on optimal dose and intervention duration should also be conducted to construct a standard paradigm for the administration of garlic supplementation. Finally, based on the current lack of management and treatment for MetS, researchers could explore other medicinal and edible products that may alleviate MetS.

## Conclusion

In conclusion, quantitative analysis of 19 RCTs (999 participants) revealed that garlic supplementation partially modulated serum lipid profile (TG, TGL, HDL), blood pressure (SBP), and anthropometric parameters (WC, BMI) of MetS. It demonstrated that garlic is a potentially beneficial medicinal food product for MetS. However, based on the current evidence, we cannot draw a robust conclusion on the beneficial extent of garlic supplementation on MetS, and further large-scale RCTs are still needed to support this conclusion.

## Supplementary Information

**Additional file 1:** **Supplementary Table S1.** PRISMA checklist of the Systematic Review and Meta-Analysis of garlic supplementation with MetS. **Supplementary Figure S1.** The results of sensitivity analysis. Supplementary Data.

## Data Availability

All data generated or analyzed during this study are included in this published article and its additional information files.
