# Supplementary material for: Effects of garlic supplementation on components of metabolic syndrome: a systematic review, meta-analysis, and meta-regression of randomized controlled trials
Source: BMC Complement Med Ther. 2023 Jul 22;23:260. doi: 10.1186/s12906-023-04038-0 (PMC10362699; doi:10.1186/s12906-023-04038-0)
Supplement: Supplementary file 1 — Additional file 1: Supplementary Table S1. PRISMA checklist of the Systematic Review and Meta-Analysis of garlic supplementation with MetS. Supplementary Figure S1. The results of sensitivity analysis. Supplementary Data. [file 12906_2023_4038_MOESM1_ESM.docx]

Supplementary Material

# Effects of garlic supplementation on components of metabolic syndrome: Systematic Review, Meta-Analysis, and Meta-regression of Randomized Controlled Trails

Zhenyue Fu1,2†, Jiayu Lv 1†, Xiya Gao1,2†, Haoran Zheng1,2, Shuqing Shi1, Xia Xu1, Bingxuan Zhang1, Huaqin Wu3, Qingqiao Song1*

^1^ Department of General Internal Medicine, Guang'anmen Hospital, China Academy of Chinese Medical Sciences, Beijing, China

^2^ Beijing University of Chinese Medicine, Beijing, China

^3^Department of Cardiology, Guang'anmen Hospital, China Academy of Chinese Medical Sciences, Beijing, China

*** Correspondence:** Qingqiao Song; [songqqbj@126.com;](mailto:songqqbj@126.com;)

# Supplementary Table

**Supplementary Table S1** PRISMA checklist of the Systematic Review and Meta-Analysis of garlic supplementation with MetS

| **Section and Topic** | **Item #** | **Checklist item** | **Location where item is reported** |
| --- | --- | --- | --- |
| **TITLE** | | |  |
| Title | 1 | The report is identified as a systematic review and meta-analysis | Page 1 |
| **ABSTRACT** | | |  |
| Abstract | 2 | The abstract includes background; information sources; the methods used to present and synthesise results; total number of included studies; results for outcomes; registration number. | Page 1 |
| **INTRODUCTION** | | |  |
| Rationale | 3 | Described in the introduction. | Page 2 |
| Objectives | 4 | Stated in the introduction. | Page 2 |
| **METHODS** | | |  |
| Eligibility criteria | 5 | We restricted the inclusion criteria based on the PICOS principle. | Page 2 |
| Information sources | 6 | We searched Pubmed, Embase, COCHRANE, Medline, and Web of science, with the time frame limited to the date of establishment to November 1, 2022, and the publication language limited to English; searched the Clinical Trials Online Registry website to find relevant trials; read references of relevant systematic reviews and reviews to reduce omissions. | Page 2 |
| Search strategy | 7 | We have submitted search strategies for all databases mentioned in this article in Supplementary data 1. | Supplementary data 1 |
| Selection process | 8 | Two investigators independently screened each record and each report retrieved on the basis of inclusion, and any disagreement was resolved by discussion. | Page 2 |
| Data collection process | 9 | Two researchers independently collected data from each report and cross-checked the results to ensure the data accuracy. Any discrepancy was resolved through discussion to reach consensus. | Page 3 |
| Data items | 10a | We collected every outcome parameter from each study. | Page 3 |
|  | 10b | The following parameters were collected from each study: 1) basic information: first author's name, nationality, institution, and year of publication; 2) baseline information: sample size, male/female ratio, mean age, health status, and baseline disease; 3) trial information: the oral form of garlic, placebo composition, dose, and duration of intervention; 4) outcomes: WC, BMI, TG, TC, LDL-c, HDL-c, FBG, SBP, DBP, adverse effects. 5) Trial process: randomization method, implementation of allocation concealment, blinded format. | Page 3 |
| Study risk of bias assessment | 11 | Two authors used the Cochrane risk of bias tool to assess methodological quality of RCTs. Each reviewer appraised bias according to  the specific content within each item, designating a low, high, or unclear risk of bias by answering yes, no or unclear. Disagreements between the two reviewers were resolved through discussion or by consulting a third author. | Page 3 |
| Effect measures | 12 | We used Cohen'd standardized mean difference (SMD) and 95%CI to evaluate the effect value. | Page 3 |
| Synthesis methods | 13a | Not mentioned. | - |
|  | 13b | For quartile data, they were transformed into mean and standard deviation format using the method developed by Hozo SP. | Page 3 |
|  | 13c | Not mentioned. | - |
|  | 13d | Data analysis was performed by Stata17 (StataCorp LP, College Station, US). Data from this study were continuous variables, and effect sizes were presented as SMD and 95% CI. Low, medium, and high levels of heterogeneity were decided by the I² statistic of 25%, 50%, and 75%. If I²>50%, significant heterogeneity was indicated, and the effect sizes were combined using a random-effects model. | Page 3 |
|  | 13e | Meta-regression and subgroup analysis were performed to detect and elucidate the sources of high heterogeneity. | Page 3 |
|  | 13f | Sensitivity analysis was performed to screen the literature impact on the robustness of the results. | Page 3 |
| Reporting bias assessment | 14 | We used contour-enhanced funnel plots, Egger's tests, and Begg's tests to detect the presence of publication bias | Page 3 |
| Certainty assessment | 15 | Not mentioned. | - |
| **RESULTS** | | |  |
| Study selection | 16a | We described the results of the search and selection process, from the number of records identified in the search to the number of studies included in the review. The literature selection process is depicted in Figure 1. | Figure 1 |
|  | 16b | We have explained this item in detail in Figure 1. | Figure 1 |
| Study characteristics | 17 | The main characteristics of the included studies in the present meta-analysis are described in Table 1. | Table 1 |
| Risk of bias in studies | 18 | Figure 2 summarizes the risk of bias for each included study according to the pre-defined criteria in Cochrane handbook. | Figure 2 |
| Results of individual studies | 19 | We used forest plots to present summary statistics for each group and effect estimates and its precision. | Figure 3,4,5,6 |
| Results of syntheses | 20a | We briefly summarise the characteristics and risk of bias among contributing studies. | Page 4 |
|  | 20b | We listed the results of all statistical syntheses, as well as each summary estimate and its precision and measures of statistical heterogeneity. | Page 4,5 |
|  | 20c | We used meta-regression and subgroup analysis to detect the possible causes of heterogeneity among study results, but no significant cause found. | Page 5 |
|  | 20d | Based on the results of our meta-analysis, we performed a sensitivity analysis for outcomes with high heterogeneity: TG, TC, LDL-C, HDL-C, SBP, and DBP. The results of sensitivity analyses showed the robustness of the results. | Page 5 |
| Reporting biases | 21 | Most of the remaining indices showed a left-right symmetric distribution in the funnel plot. Egger's and Begg's tests (p<0.05) suggested the presence of publication bias of HDL. | Page 6 |
| Certainty of evidence | 22 | We assessed the certainty (or confidence) in the body of evidence for each outcome assessed. | Page 6 |
| **DISCUSSION** | | |  |
| Discussion | 23a | In the context of other evidence, we provide a general interpretation of the effect of garlic supplementation on metabolic components. | Page 6 |
|  | 23b | We discuss the limitations of the evidence included in the review. | Page 8 |
|  | 23c | Not mentioned. | - |
|  | 23d | Based on strict inclusion and exclusion criteria, we screened 19 RCTs with 999 participants. Quantitative analysis of these RCTs revealed that garlic supplementation partially modulated serum lipid profile (TG, TGL, HDL), blood pressure (SBP), and anthropometric parameters (WC, BMI) of metabolic syndrome. However, based on the current evidence, we cannot draw a solid conclusion on the beneficial extent of garlic supplementation on metabolic syndrome. | Page 8 |
| **OTHER INFORMATION** | | |  |
| Registration and protocol | 24a | PROSPERO (<https://www.crd.york.ac.uk/PROSPERO/>) ID: CRD42022373228 | Page 2 |
|  | 24b | The protocol was not prepared. | - |
|  | 24c | Not mentioned. | - |
| Support | 25 | The authors declare that the research was conducted in the absence of any commercial or financial relationships that could be construed as a potential conflict of interest. This study is supported by the Scientific and technological innovation project of China Academy of Chinese Medical Sciences (CI2021A01603). | Page 9 |
| Competing interests | 26 | The research was conducted in the absence of any commercial or financial relationship that could be construed as a potential conflict of interest. | Page 9 |
| Availability of data, code and other materials | 27 | The original contributions presented in the study are included in the article/Supplementary Material. Further inquiries can be directed to the corresponding authors. | Page 9 |

*From:*  Page MJ, McKenzie JE, Bossuyt PM, Boutron I, Hoffmann TC, Mulrow CD, et al. The PRISMA 2020 statement: an updated guideline for reporting systematic reviews. BMJ 2021;372:n71. doi: 10.1136/bmj.n71

# Supplementary Figure

##
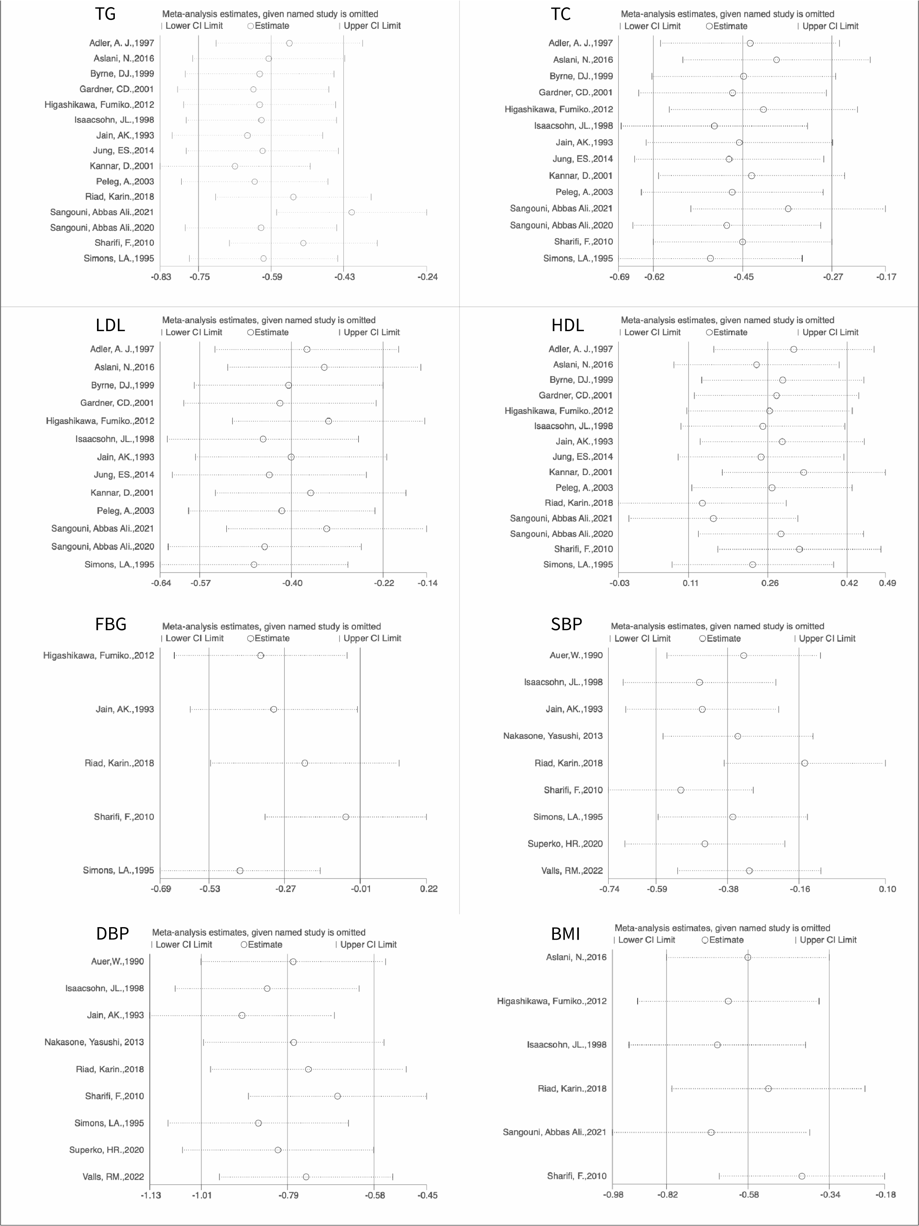


**Supplementary Figure S1 The results of sensitivity analysis.**

# Supplementary Data

**Supplementary Data 1 Retrieval strategy: CORCHANE as an example**

#1 MeSH descriptor: [Garlic] explode all trees

#2 (allicin):ti,ab,kw OR (Allium sativum):ti,ab,kw

#3 #1 OR #2

#4 MeSH descriptor: [Metabolic Syndrome] explode all trees

#5 (Syndrome X):ti,ab,kw OR (Metabolic X Syndrome):ti,ab,kw OR (Syndrome, Cardiometabolic):ti,ab,kw

#6 #4 OR #5

#7 MeSH descriptor: [Hypertension] explode all trees

#8 High Blood Pressure

#9 MeSH descriptor: [Blood Pressure] explode all trees

#10 #7 OR #8 OR #9

#11 MeSH descriptor: [Hyperlipidemias] explode all trees

#12 (Lipidemia):ti,ab,kw OR (Lipemia):ti,ab,kw OR (Hyperlipidemia):ti,ab,kw

#13 MeSH descriptor: [Lipids] explode all trees

#14 MeSH descriptor: [Cholesterol] explode all trees

#15 #11 OR #12 OR #13 OR #14

#16 MeSH descriptor: [Blood Glucose] explode all trees

#17 MeSH descriptor: [Insulin Resistance] explode all trees

#18 (Insulin Sensitivity):ti,ab,kw OR (Sensitivity, Insulin):ti,ab,kw OR (Resistance, Insulin):ti,ab,kw

#19 #16 OR #17 OR #18

#20 MeSH descriptor: [Waist Circumference] explode all trees

#21 MeSH descriptor: [Body Mass Index] explode all trees

#22 #6 OR #10 OR #15 OR #19 OR #20 OR #21

#23 (randomized):ti,ab,kw OR (controlled):ti,ab,kw

#24 #3 AND #22 AND #23
